# Prevalence of *Ehrlichia* spp. in dogs and ticks in Hainan Province, China

**DOI:** 10.1186/s12917-024-04434-9

**Published:** 2025-04-03

**Authors:** Haiyue Zu, Zhilong Xiang, Xiaoming Zhang, Qiyuan Cao, Yang Lin, Zhu Ying, Biswajit Bhowmick, Hengtao Xiang, Qian Han, Jinhua Wang

**Affiliations:** 1 School of Tropical Agriculture and Forestry, Hainan University, Danzhou, 571737 China; 2 School of Life and Health Sciences, Hainan Province Key Laboratory of One Health, Collaborative Innovation Center of One Health, Hainan University, Haikou, Hainan 570228 China; 3 Hainan International One Health Institute, Hainan University, Haikou, Hainan 570228 China; 4 Baoting Li and Miao Autonomous County Animal Husbandry and Fishery Service Center, Baoting, Hainan, 572300 China; 5 National Animal Protozoa Laboratory, College of Veterinary Medicine, China Agricultural University, Beijing, 100083 China; 6 Department of Veterinary Microbiology and Pathology, College of Veterinary Medicine, Washington State University, Pullman, WA USA

**Keywords:** Dog, *Ehrlichia*, Tick, PCR, Phylogenetic analysis

## Abstract

**Background:**

*Ehrlichia* spp. are a group of intracellular parasitic bacteria primarily transmitted by ticks. They exhibit a wide global distribution and can infect a diverse range of mammals, including humans, underscoring their immense public health significance.

**Results:**

Among 631 ticks examined, all were identified as belonging to the *Rhipicephalus linnaei*; of these, 63 (9.98%) out of 631 ticks tested positive for *Ehrlichia canis*.Additionally, 140 (11.08%) out of 1264 dog blood samples were positive for *E. canis*. Notably, *Ehrlichia ewingii* and *Ehrlichia chaffeensis* were not detected. The prevalence of *Ehrlichia* infection in dogs was associated with factors such as age, breed, dewormer use, tick infestation, and living environment while displaying no association with the dog’s gender.

**Conclusions:**

In Hainan Province, *Rhipicephalus linnaei* is the dominant tick species infecting dogs. Dogs are vulnerable to *Ehrlichia* infection, particularly rural and stray dogs, suggesting the need for a targeted control strategy.

## Background

Ehrlichiosis is a tick-borne bacterial zoonotic disease caused by *Ehrlichia* spp. *Ehrlichia* spp. are Gram-negative obligate intracellular bacteria that invade the blood cells of host animals and belong to the genus *Ehrlichia*, family Anaplasmataceae. While *Ehrlichia’*s natural hosts are foxes, coyotes, and jackals, it can infect various vertebrates, including dogs, horses, cows, sheep, rats, and humans. Currently, there are six reported species of *Ehrlichia*: *E. canis*, *E. chaffeensis*, *E. ewingii*, *Ehrlichia minasensis*, *Ehrlichia muris*, and *Ehrlichia ruminantium* [1]. In 1925, Cowdry et al. was the first to discover *Ehrlichia ruminantium* in cattle; a decade later, Donatien and Lestoquard described *E. canis* in Algerian dogs. *Ehrlichia* disease constitutes a serious threat to livestock breeding and pet health. In the late 20th century, *Ehrlichia* was recognized as a zoonotic human pathogen of public health importance since the discovery of *E. chaffeensis* and *E. ewingii*, which are pathogenic to humans. Currently, no commercial vaccines are available to protect against infections with *Ehrlichia* [2, 3].

Canine ehrlichiosis is a prevalent tick-borne disease affecting dogs worldwide. Three main species of *Ehrlichia* can infect dogs: *E. canis*, *E. chaffeensis*, and *E. ewingii* [4]. *E. canis* is the most prevalent and significant species in dogs. It was also the first strain of *Ehrlichia* to be discovered, primarily infecting canine monocytes and causing Canine Monocytic Ehrlichiosis. *E. chaffeensis* primarily infects humans by parasitizing peripheral circulating monocytes, causing human monocytic Ehrlichiosis (HME) and dogs [5]. *E. ewingii* primarily infects canine Peripheral Blood Neutrophils (PBNs), causing Canine Granulocytic Ehrlichiosis (CGE), and humans, causing human Ehrlichiosis. *E. ewingii* was first discovered in 1991 in canine blood in the United States and is phylogenetically closely related to *E. canis* and *E. chaffeensis* [6].

Clinical signs in dogs naturally infected with *Ehrlichia* and suffering from the disease mainly include fever or hypothermia due to excessive hematocrit, depression or lethargy, anorexia, generalized lymph node enlargement, splenomegaly, pale mucous membranes, hemorrhagic tendencies and ocular abnormalities [7]. However, in veterinary clinics, the symptoms caused by *Ehrlichia* infections may vary, depending on many factors such as the state of the host’s immune system, the virulence of the infecting strain, and the presence of co-infections with other mosquito/tick/flea-borne diseases.

*Ehrlichia* infections in humans result in HME, an acute febrile illness characterized by nonspecific clinical manifestations. The main symptoms include fever, myalgia, arthralgia, fatigue, headache, nausea, and vomiting. Most cases of HME are caused by *E. chaffeensis*, with a few caused by *E. ewingii* [2].

Ticks, including various hard tick species, are the vector organisms that transmit *Ehrlichia*. *E. canis* is the most prevalent and damaging dog pathogen, primarily transmitted by *Rhipicephalus linnaei* (the brown dog tick, previously named *R. sanguineus sensu lato*) [8].

*E. chaffeensis* is mainly transmitted by *Amblyomma americanum*, *Dermacentor variabilis*, and *Ixodes pacificus* [9]. Similarly, *E. ewingii*, like *E. chaffeensis*, is primarily transmitted by *A. americanum* [10].

*canis* has a global distribution and has been reported in several countries across Africa, Europe, Asia, and the Americas, with a higher prevalence in the tropics and subtropics [11]. On the other hand, *E. chaffeensis* and *E. ewingii* are primarily located in the southeastern and south-central United States, with *E. ewingii* also reported in Africa and Asia [12]. Presently, Ehrlichiosis in dogs and humans caused by *E. chaffeensis* and *E. ewingii* has become an endemic disease in the United States. In China, *E. canis* and *E. chaffeensis* have been detected in ticks and dogs [9, 13]. *E. chaffeensis* has been reported only from Xinjiang in dogs [14]. *E. ewingii* has not been reported in either ticks or dogs. Notably, there are no reports of *Ehrlichia infections* in dogs and ticks in Hainan province.

In a global context where populations and economies are expanding, the number of pet owners, particularly dog owners, continues to rise. While dogs serve as loyal companions, they pose a potential public health risk by increasing the prevalence of tick-borne diseases in humans. Ticks, acting as vectors for transmitting these diseases, have a high potential to transfer pathogens from the body surface of dogs to humans during human-canine contact, leading to tick-borne disease infections in humans.

Hainan province, situated in the southernmost part of China, features a tropical climate characterized by year-round warmth and humidity, providing an ideal environment for the growth and reproduction of ticks. Consequently, it is considered a high-prevalence area for tick-borne diseases. However, despite these conditions, the prevalence of *Ehrlichia* infection in dogs and ticks has not been reported in Hainan province, necessitating the collection of additional comprehensive data to cover the region. The study seeks to contribute new epidemiological data on canine vector-borne diseases in Hainan province and to establish a database for the prevention and treatment of tick-borne diseases in both dogs and humans in the region. This study used PCR detection based on the tick and Ehrlich 16 S RNA gene (Ribosomal RNA) to identify different species. 16 S RNA gene is conserved within species that would indicate a gene that is constitutively expressed and can thus be targeted in studies to determine the prevalence of the bacteria in the analyzed vector or host samples.

## Methods

### Samples collection

**Fig. 1:**
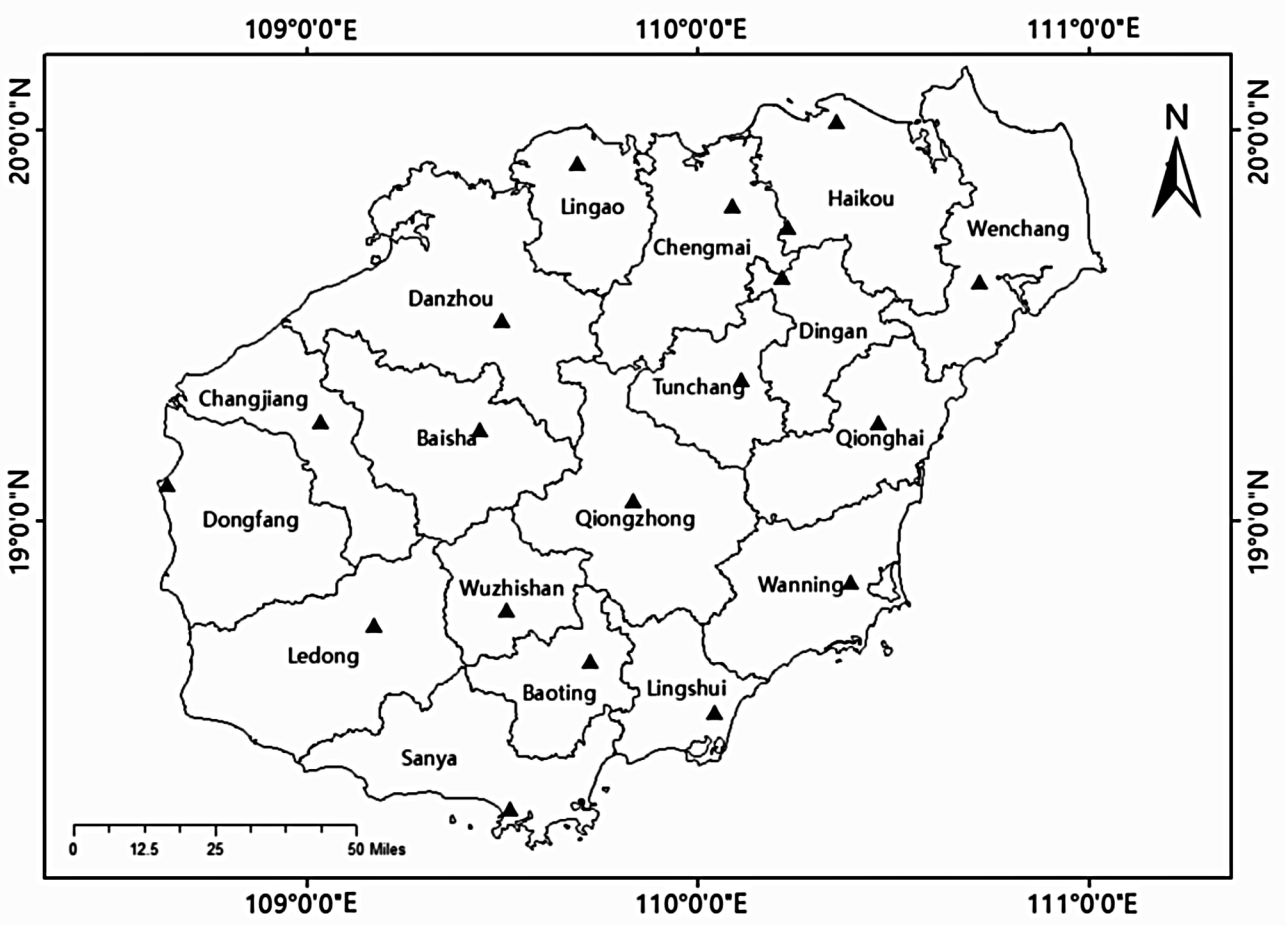
Map of Hainan showing locations where the samples were collected

Between March 2019 and December 2023, 631 ticks were collected from dogs located in four cities and counties (Baisha, Ding’an, Haikou, and Lingshui) in Hainan province, China. One thousand two hundred sixty-four canine blood samples were collected from dogs in 18 cities and counties in Hainan province (Fig. 1).

Blood collection (1 ~ 2 mL) was performed from the cephalic vein and put into tubes with anticoagulant (ethylene diamine tetraacetic acid [EDTA]). During blood collection, dogs were thoroughly examined for ectoparasites. Ticks were gently collected using forceps and placed in labeled plain sterile sample bottles containing 75% alcohol.

### DNA extraction

All collected samples were transferred to the laboratory at 4 °C. Within 24 h of sample collection, DNA extraction was conducted. According to the manufacturer’s instructions, genetic DNA was extracted using 100 µL of blood sample (Ezup Column Blood Genomic DNA Purification Kit, Sangon Biotech, China). Individual ticks were rinsed twice with 75% alcohol and sterile distilled water, and air dried for 5 min on sterile paper. The Ezup Column Animal Genomic DNA Purification Kit (Sangon Biotech, China) was utilized in accordance with the manufacturer’s protocol to perform ticks DNA extraction. The individual tick specimens were then placed in a 2 mL microcentrifuge tube filled with steel beads ( 0.1 and 3 mm in diameter), together with 180 µL of Buffer ACL and 20 µL of proteinase K, before homogenizing while shaking for 3 min in a tissue grinder at 60 HZ for extracting DNA. The extracted tick and canine blood DNA samples were stored at − 20 °C for further experimental use.

### PCR amplification

PCR was performed using primers derived from the 16 S RNA gene. These primers target a conserved region within the 16 S RNA gene for ticks and amplification results in a 460 bp PCR product. Subsequently, the same PCR primers were used as sequencing primers to determine the sequence of the amplified PCR products. 16 S F(CTGCTCAATGATTTTTTAAATTGCTGTGG) and 16 S R(CCGGTCTGAACTCAGATCAAGT), were used for the amplification of *16 S rRNA* gene fragment of ticks (460 bp) [15]. PCR mixture contained 12.5 µL of 2×Taq Plus Master MixII (Dye Plus) (Vazyme Biotech Co., Ltd), 10.5 µL of ddH2O, 0.5 µL of each primer (10pmol/µL), and 1 µL of extracted DNA(~ 20ng) in a volume of 25 µL. The PCR amplifications were performed in a Perkin-Elmer model 480 thermal cycler, using the following protocol: preheating at 95 °C for 5 min, followed by 35 cycles of 95 °C for 30 s, 58 °C for 30 s, and 72 °C for 45 s, and then a final extension at 72 °C for 5 min. Both a negative control (ddH2O) and a positive control were included in each set of amplifications. PCR products were examined on 1% agarose gel stained with 0.1% GoldenView using a Quick-Load 2 kb DNA Ladder marker (TAKARA BIO, Inc. China), visualized under the Gel Doc XR Imaging system (Bio-Rad Laboratories, Inc.). Subsequently, the same PCR primers were used as sequencing primers to determine the sequence of the amplified PCR products.

### Nested PCR amplification

Nested PCR was performed using primers derived from the 16 S RNA gene. These primers target a conserved region within the 16 S RNA gene for *E. canis*, *E. chaffeensis*, and *E. ewingii*, and amplification results in a 396 bp PCR product [16]. Outer primers, ECC(AGAACGAACGCTGGCGGCAAGC) and ECB(CGTATTACCGCGGCTGCTGGCA) were used for the amplification of all *Ehrlichia* spp.(477 bp). Inner primers, ECAN5(CAATTATTTATAGCCTCTGGCTATAGGA) and HE3(TATAGGTACCGTCATTATCTTCCCTAT), were used for the *E. canis*-specific amplifications (396 bp), HE1(CAATTGCTTATAACCTTTTGGTTATAAAT) and HE3, were used for the *E. chaffeensis*-specific amplifications (396 bp), EE52(CGAACAATTCCTAAATAGTCTCTGAC) and HE3, were used for the *E. ewingii*-specific amplifications (396 bp) (Table 1).The same set of primers from the second round was also used for sequencing separately. PCR mixture contained 12.5 µL of 2×Taq Plus Master MixII (Dye Plus) (Vazyme Biotech Co., Ltd), 10.5 µL of ddH2O, 0.5 µL of each primer (10pmol/µL), and 1 µL of extracted DNA (~ 20ng) in a volume of 25 µL. The PCR amplifications were performed in a Perkin-Elmer model 480 thermal cycler, using the following protocol: reactions with primers ECC and ECB consisted of preheating at 94 °C for 3 min, followed by 30 cycles of 94 °C for 1 min, 65 °C for 2 min, and 72 °C for 2 min, and then a final extension at 72 °C for 5 min. Reactions with species-specific primers consisted of preheating at 94 °C for 3 min, followed by 40 cycles of 94 °C for 1 min, 55 °C for 2 min, and 72 °C for 1.5 min, and then a final extension at 72 °C for 5 min. Both a negative control (ddH2O) and a positive control were included in each set of amplifications. PCR products were examined on 1% agarose gel stained with 0.1% GoldenView using a Quick-Load 2 kb DNA Ladder marker (TAKARA BIO, Inc. China), visualized under the Gel Doc XR Imaging system (Bio-Rad Laboratories, Inc.). Subsequently, the same PCR primers were used as sequencing primers to determine the sequence of the amplified PCR products.

**Table 1:** Prevelence of *Ehrlichia* spp. infection in dogs and ticks of Hainan province

Species	City/County	No. of samples tested	No. of E. canis PCR positive samples (%)
Canis	Baisha	11	2 (18.18)
	Baoting	24	1 (4.17)
	Changjiang	15	2 (13.33)
	Chengmai	16	3 (18.75)
	Danzhou	73	5 (6.85)
	Dingan	327	84 (25.69)
	Dongfang	21	2 (9.52)
	Haikou	533	22 (4.13)
	Ledong	16	3 (18.75)
	Lingao	12	0
	Lingshui	14	4 (28.57)
	Qionghai	34	1 (2.94)
	Qiongzhong	19	3 (15.79)
	Sanya	43	2 (4.65)
	Tunchang	30	5 (16.67)
	Wanning	18	0
	Wenchang	46	1 (2.17)
	Wuzhishan	12	0
	Total	1264	140 (11.08)
Tick	Baisha	57	9 (15.79)
	Dingan	440	51 (11.59)
	Haikou	31	0
	Lingshui	103	3 (2.91)
	Total	631	63 (9.98))

### Sequencing and phylogenetic analysis

All amplified PCR products were purified using a commercial DNA gel purification kit (Sangon Biotech, China) according to the manufacturer’s instructions and then sent to Sangon Biotech and Bio-engineering in Shanghai for DNA sequencing. All obtained DNA sequences were compared with those available in the GenBank database using BLAST (http://blast.ncbi.nlm.nih.gov/Blast.cgi) to determine the identity of the DNA sequences. Phylogenetic and molecular evolutionary analysis was performed using the neighbor-joining method with 1000 replicates for bootstrap analysis in MEGA XI. The sequences obtained in this study were deposited into GenBank with the accession numbers: *R. linnaei* (PP087098-PP087103), *E. canis* (PP087090-PP087092, PP087094- PP087096).

### Analysis of risk factors associated with *Ehrlichia* infection

Risk factors affecting *Ehrlichia* infection were statistically analyzed using SPSS V26.0 software. Whether a dog was infected with *Ehrlichia* was used as the dependent variable, and the sex (female or male), age (<1 year or ≥ 1 year), breed (pure Breed or mixed breed), tick infestation (present or absent), anthelmintic (used or unused). The environment (urban, animal shelter, or rural) was analyzed by regression using a binary logistic model. The variable was considered to be statistically significant at *P* < 0.05. Confidence intervals were set at 95% for the dominance ratio (OR).

## Results

### PCR results for *Ehrlichia* spp. in dogs and tick

**Fig. 2:**
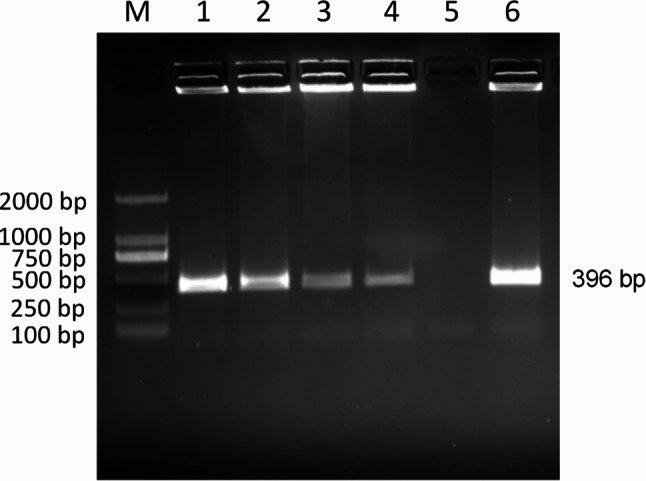
PCR amplification of *E. canis* 16 S rRNA gene. Lane M: DL-2000 DNA marker; Lanes 1–4: Test sample; Lane 5: Negative control; Lane 6: Positive control

**Fig. 3:**
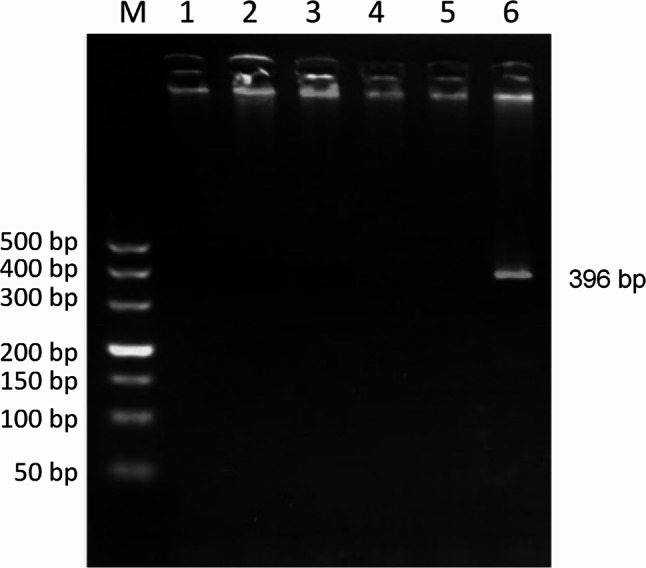
PCR amplification of *E. chaffeensis* 16 S rRNA gene. Lane M: DL-2000 DNA marker; Lanes 1–4: Test sample; Lane 5: Negative control; Lane 6: Positive control

**Fig. 4:**
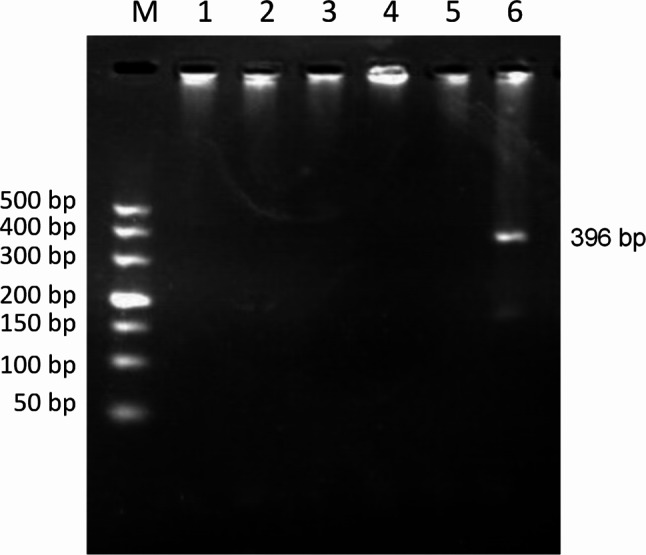
PCR amplification of *E. ewingii* 16 S rRNA gene. Lane M: DL-2000 DNA marker; Lanes 1–4: Test sample; Lane 5: Negative control; Lane 6: Positive control

**Fig. 5:**
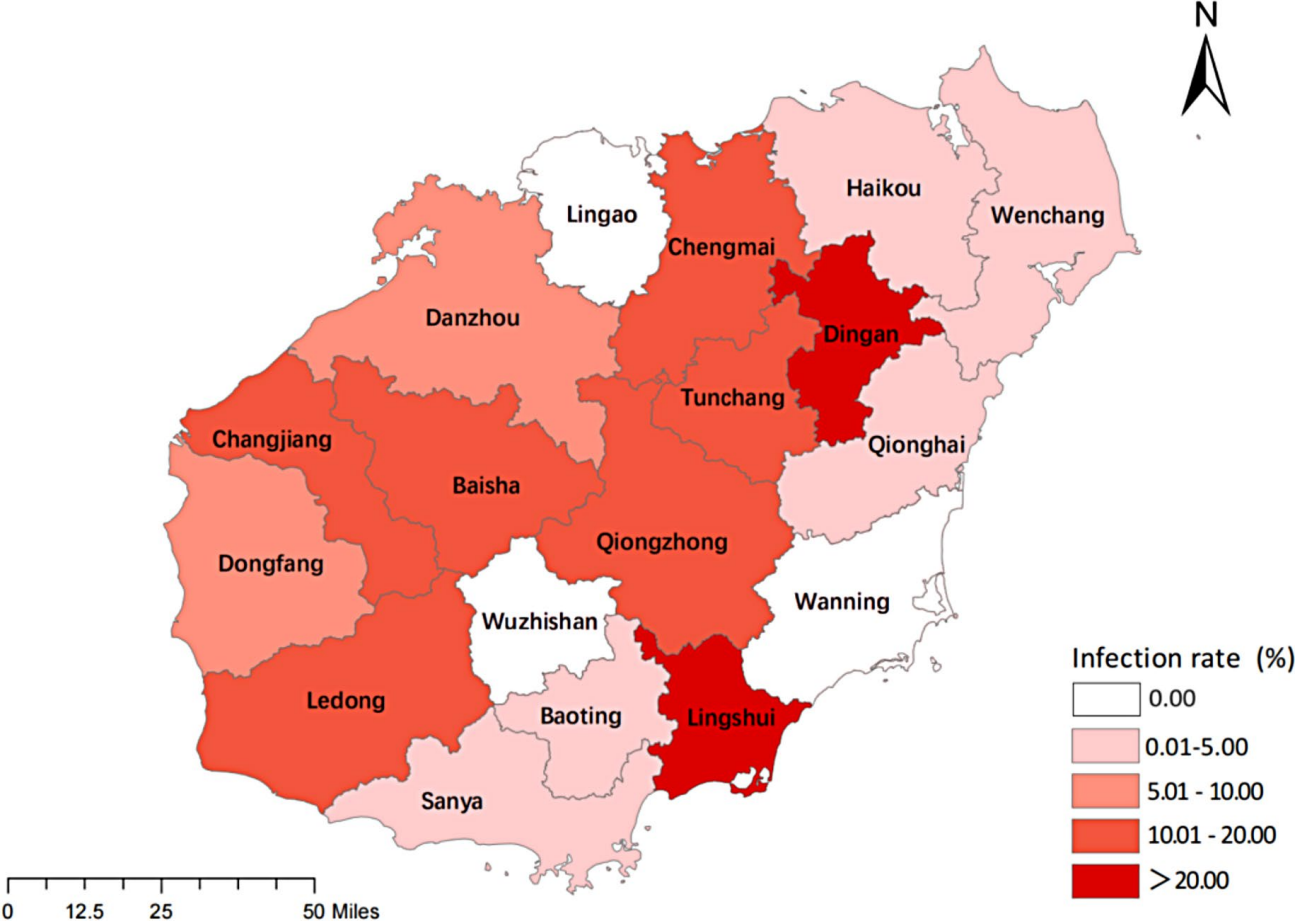
Prevalence of *E.canis* infection in dogs from Hainan island. Distribution of *E.canis* infections in dogs from Hainan island based on *16srRNA* gene detection via PCR.The colour shade reflects prevalence range (percentages)

PCR detection of *Ehrlichia* spp. was performed on 1264 canine blood DNA samples, showing that 140 (11.08%) of the samples were positive for *Ehrlichia* spp. PCR results showed that the amplification of *E. canis* was positive (396 bp)(Fig. 2), and the amplification results of *E. chaffeensis* (Fig. 3) and *E. ewingii* (Fig. 4) were negative. Sequencing confirmed all positive samples as *E. canis*, with no detection of *E. chaffeensis* and *E. ewingii*. Table 1; Fig. 5 illustrate the prevalence of *Ehrlichia* spp. infeciton in Hainan province, respectively. The highest rates were observed in Lingshui (28.57%) and Ding’an (25.69%), exceeding 20%. Additionally, Chengmai (18.75%), Ledong (18.75%), and Baisha (18.18%) demonstrated high infection rates. In contrast, Lingao, Wanning, and Wuzhishan yielded no positive detections.

Similarly, all 631 tick DNA samples were tested by PCR for *Ehrlichia* spp. The positive samples were subjected to sequencing. 63 (9.98%) samples were tested positive for *Ehrlichia* spp. and identified as *E. canis* by sequencing, whereas *E. chaffeensis* and *E. ewingii* were not detected. Notably, 9 *E. canis* positives were identified in Baisha, with the highest infection rate (15.79%), while no positive detections were reported in Haikou (Table 1).

### Species identification and phylogenetic analysis of ticks

**Fig. 6:**
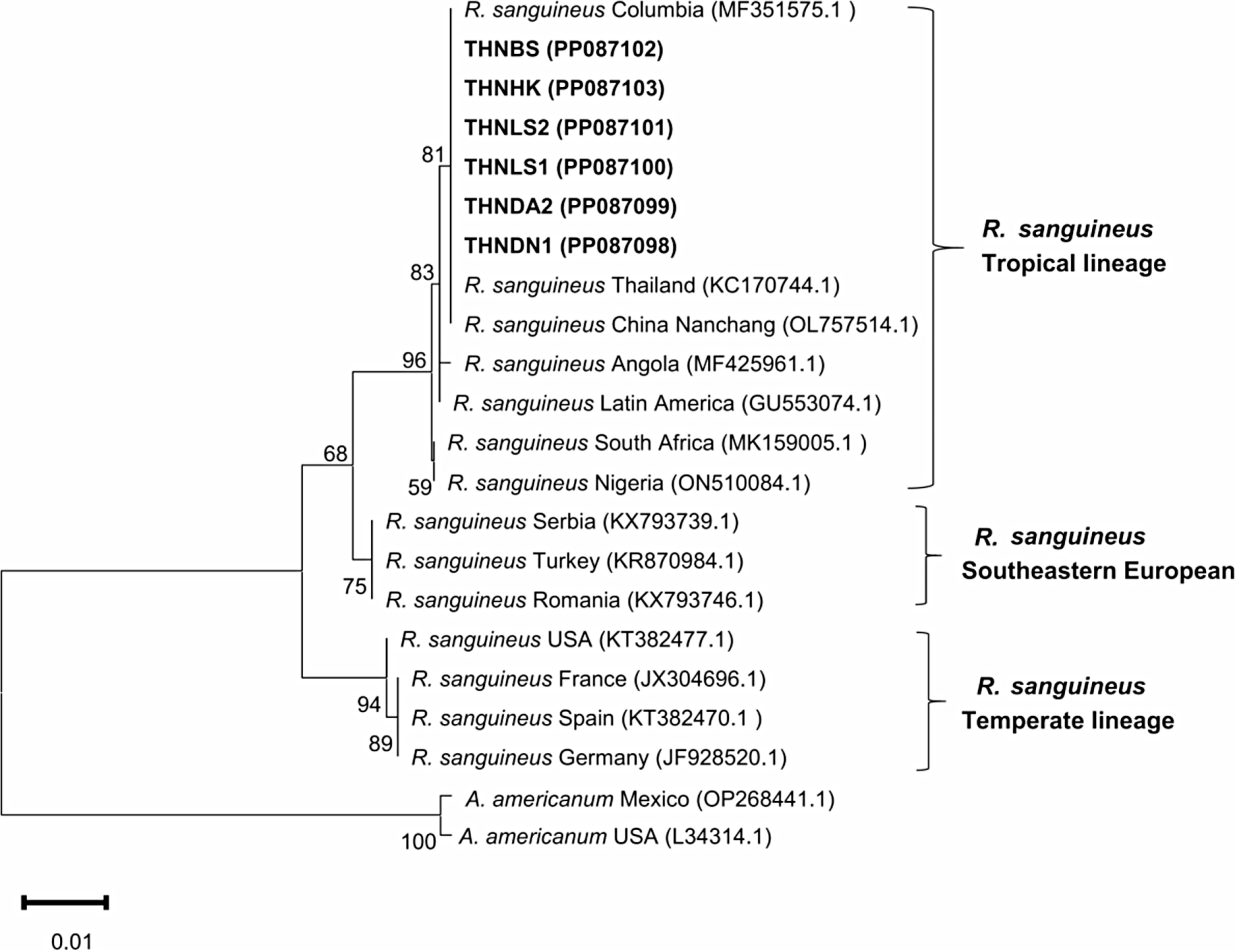
Neighbor-joining (NJ) phylogenetic tree of tick based on the 16 S rRNA gene sequences. Evolutionary analyses were conducted in MEGA XI. Bootstrap values (1000 replications) are shown on the branches. Sequences generated from this study are in bold font

After PCR amplification, *16 S rRNA* gene sequencing was performed on all 631 tick DNA samples collected. BLASTN analysis of the results confirmed the identification of the collected ticks as *R.linnae*(*R. sanguineus s.l.*). A phylogenetic tree was constructed for the six *R. linnaei* sequences (GenBank: PP087098-PP087103) based on the *16 S rRNA* gene using MEGA-XI software (Fig. 6). The 16 S rRNA genes of all haemaphticks in this study were clustered in a tropical lineage branch closely related to ticks from Columbia, Thailand, and China Nanchang. *R. sanguineus* ticks in the southeast European lineage showed a sister relationship with the tropical lineage. The sequence of temperate lineages formed a distinct clade away from the tropical lineages.

### Phylogenetic analysis of *E. canis*

**Fig. 7:**
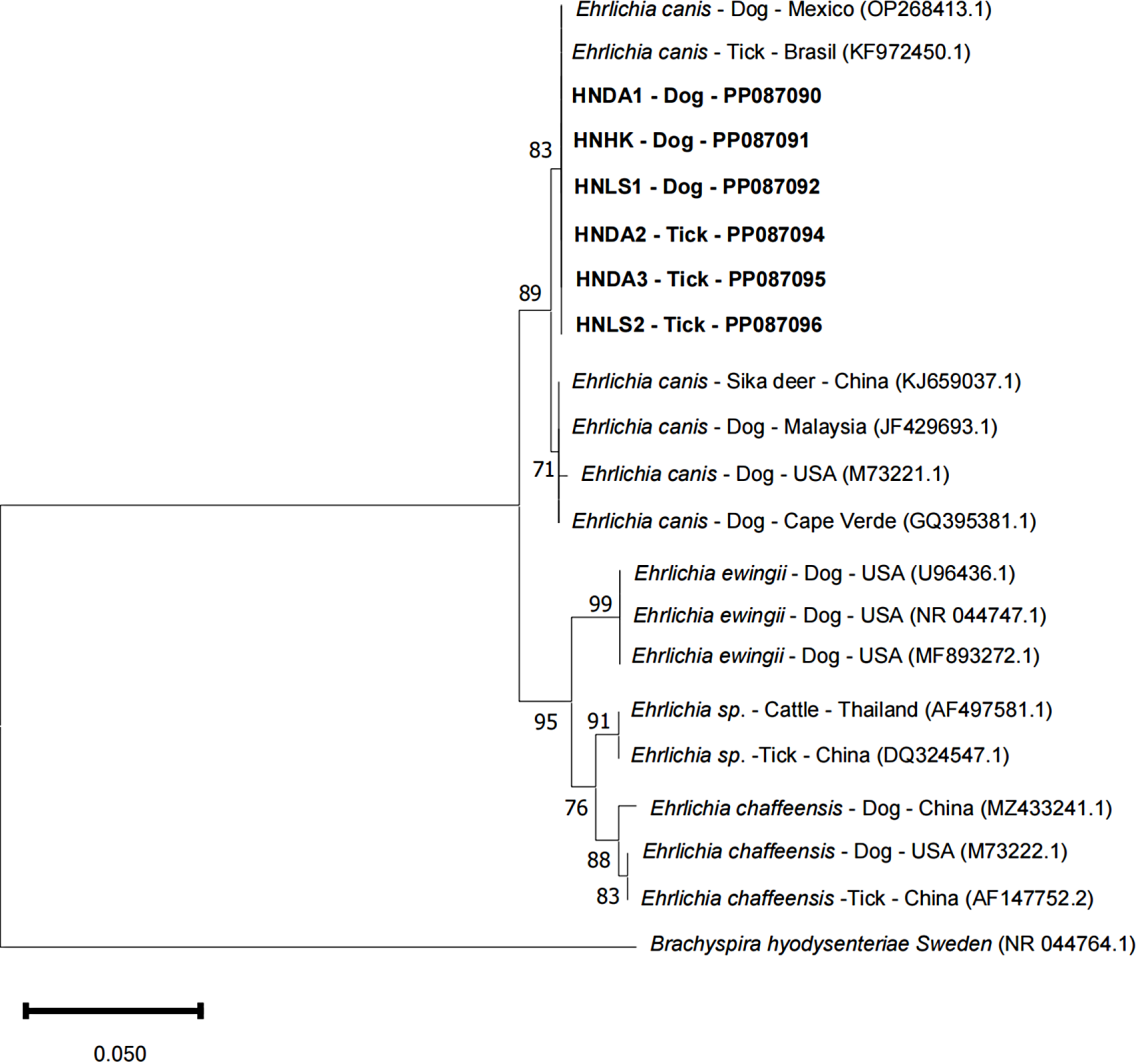
Neighbor-joining (NJ) phylogenetic tree of *E .canis* based on the *16 S rRNA* gene sequences. Evolutionary analyses were conducted in MEGA XI. Bootstrap values (1000 replications) are shown on the branches. Sequences generated from this study are in bold font

An analysis of the *E. canis* sequencing products based on *16 S rRNA* regions obtained in this study (GenBank: PP087090-PP087092, PP087094-PP087096) revealed 99–100% identity between *E. canis* from ticks and dogs. In the phylogenetic tree based on *16 S rRNA* genes, it was observed that all *E. canis* sequences from Hainan province formed a distinct closely related to *E. canis* sequences detected in Mexico (OP268413.1) and Brazil (KF972450.1) (Fig. 7), These sequences were clearly differentiated from other *Ehrlichia* species, highlighting their genetic distinction.

### Risk factors associated with *E. canis* infection

A binary logistic model was utilized to analyze the effects of sex, age, breed, tick infestation, anthelmintic use, and husbandry environment on the prevalence of *E. canis* infection. The results of the analyses are presented in Table 2. The prevalence of *Ehrlichia* infection in dogs is correlated with age, breed, dewormer use, tick infestation, and living environment, with significant differences (*P* < 0.05) while displaying no association with the gender of the dog(*P* = 0.370>0.05).

**Table 2:** Risk factors associated with the prevalence of *E .canis* infection

Variables		No. of dogs examined (*n* = 1264)	No. Positive (%)	OR (95% CI)	*P*-value
Gender	Male	667	74 (11.09)	0.996 (0.699–1.420)	0.370
	Female	597	66 (11.06)	Reference	
Breed	Pure Breed	525	34 (6.48)	Reference	<0.001
	Mixed breed	739	106 (14.34)	2.418 (1.615–3.622)	
Age	<1	461	39 (8.46)	1.557 (1.055–2.296)	0.026
	≥ 1	803	101 (12.58)	Reference	
Tick infestation	Present	102	32 (31.37)	4.461 (2.809–7.087)	<0.001
	Absent	1162	108 (9.29)	Reference	
Anthelmintic	Used	475	37 (7.79)	Reference	0.004
	Unused	789	103 (13.05)	1.777(1.198–2.637)	
Living environment	Urban	285	11 (3.86)	Reference	
	Animal shelter	301	53 (17.61)	5.323 (2.719–10.421)	0.001
	Rural	678	76 (11.21)	3.145 (1.645–6.031)	0.001

## Discussion

Tick-borne diseases hold significant importance in dog and human health and have increasingly become a global concern. As companion animals, dogs spend considerable time in close proximity to humans, heightening the risk of tick bites in humans. Moreover, the globalization of the pet trade facilitated by social media and other trading platforms has paved the way for more effortless international mobility for pets, consequently allowing tick-borne diseases to pose threats in new regions.

In this study, we delved into the prevalence of *Ehrlichia* spp. in ticks and dogs across specific areas of Hainan province, China. Ticks extracted from the body surface of dogs in Tunchang, Ding’an, Ledong, and Baisha underwent molecular testing for species identification. All ticks were identified as *R. linnaei*. *R. linnaei* stands out as a predominant tick species in dogs in China. For instance, in a 2017 nationwide study on tick-borne diseases in pet dogs across 20 Chinese cities, out of 1,550 ticks collected, 1,058 (68.3%) were identified as *R. linnae*i [17]. Known for its adaptability and global distribution, *R. linnaei* ranks as one of the most prevalent parasitic tick species infesting dogs worldwide. The study’s findings homed in on a sole tick species, which could be attributed to *R. linnaei* being a tropical tick species thriving in the hot and humid climatic conditions of Hainan Island. Alternatively, the monoculture of tick species may also stem from restrictions in sampling sites and sample numbers. Thus, expanding the sampling sites and sample sizes is imperative for a more comprehensive understanding of parasitic tick species infesting canines.

Current genetic studies suggest the existence of four potential lineages within *R. sanguineus* based on geographical location: the temperate lineage (*R. sanguineus s.l.*) and tropical lineages (*R. sanguineus s.s*., *R. linnaei* ), southeastern Europe and Afrotropical lineages (*R. afranicus*) [18, 19]. This study’s phylogenetic analysis of 16 S rRNA genes identified *R. sanguineus* in Hainan Island as clustered into one tropical lineage clade. This is consistent with the geography of Hainan Island.

Moreover, our investigation detected 63 (9.98%) *E. canis*-positive samples among 631 ticks and 140 (11.08%) *E. canis* infection cases among 1,264 dogs. A comparative analysis of the *16 S rRNA* gene sequences of *E. canis* in tick samples and dogs highlighted a high level of similarity, reinforcing the notion of *R. linnaei* being a vector for this pathogen. The positivity rates of *E. canis* in dogs and ticks in Hainan province were similar to those reported in some regions of China. In Xinjiang, the prevalence was 10.2% in ticks and 12.12% in dogs [20]. In south-central and southwestern China, the positivity rate in ticks was 11.59% [21]. Notably, the infection rate of *E. canis* in Hainan province surpassed that of economically developed regions such as Beijing (1.49%) and Jiangsu (4.69%) [13]. Economic development and urbanization levels might underpin the variation in *E. canis* infection rates. Additionally, only *E. canis*, but not *E. ewingii* and *E. chaffeensis*, was detected in this study. Additionally, only *E. canis*, and not *E. ewingii* and *E. chaffeensis*, was detected in this study, attributable to the primary transmission of *E. canis* by *R. linnaei*, whereas *E. ewingii* and *E. chaffeensis* are chiefly transmitted by *A. americanum*, a tick species primarily found in the United States and Canada and rarely reported in China [22, 23].

In this investigation, *E. canis* was detected in dogs in Haikou, with a positivity rate of 4.13% (22/533), which stood below the average infection rate observed in Hainan province. Notably, *E. canis* was not detected in ticks in Haikou. The rationale behind this absence may be that most dogs in Haikou originated from pet hospitals and were primarily urban pets, thereby facing a reduced risk of tick exposure. Additionally, the dog owners displayed a good understanding of *parasite prevention* practices. It was noted that sure dog owners opted to manually remove ticks from their pets upon discovery, delaying veterinary visits until unusual symptoms manifested, consequently limiting the tick samples collected to a mere 31, rendering the test results non-representative.

The incidence of *E. canis* infection was more prevalent in adult dogs (≥ 1 year old) compared to young dogs (<1 year old). This outcome aligns with several existing reports [11, 24]. This pattern may be ascribed to the increased likelihood of adult dogs encountering ticks and acquiring *E. canis* over their lifetime. Furthermore, the gradual decline in immunity as dogs age could contribute to the elevated infection rates observed in older dogs.

In this study on the risk factors of *E. canis* infection, there is a correlation between the breed of dogs and the prevalence of infection, noting a higher prevalence among mixed-breed dogs than purebred dogs. However, this disparity does not necessarily imply that mixed-breed dogs are inherently more susceptible to infection than purebred ones. In China, purebred dogs are often more expensive and owned by families with better financial situations and higher awareness of *parasite prevention* practices. Mixed-blood dogs, especially Chinese Field Spaniels, dominate the rural dog population in Hainan province due to lower breeding costs and inadequate parasite prevention awareness among their owners. The difference in living conditions and parasite control contributes to the elevated positivity rate in mixed-breed dogs. In a serological examination for canine ehrlichiosis in three rural areas of Brazil, the results indicated a higher positivity rate in non-purebred dogs compared to purebred ones, aligning with the current study’s findings [25]. Similarly, an experimental study involving German shepherds and Beagles simultaneously infected with *E. canis* demonstrated a weaker cell-mediated immune response in German shepherds, rendering them more susceptible to infection than Beagles [26]. While the breed of the dog appears to be a risk factor for *Ehrlichia* infection, further controlled experimental investigations are warranted to explore potential significant differences in infection prevalence among specific dog breeds.

When analyzing dogs residing in diverse housing environments, urban dogs exhibited the lowest infection rate (3.86%), whereas shelter dogs had the highest (17.61%), followed by rural dogs (11.21%). Urban dogs typically have restricted movement, limited tick exposure, and owners with heightened *parasite prevention* awareness. In contrast, with their unrestricted or semi-restricted lifestyle, rural dogs are usually unrestricted and semi-restricted with activities that include grass and come in contact with tick habitats like grass and shrubs, heightening their risk of tick bites. Moreover, rural dog owners often lack *parasite prevention* awareness, contributing to a higher infection rate among rural dogs. Stray dog shelters in remote jungle areas of Hainan province present a setting where dogs are prone to tick exposure due to high tick density, fostering easy transmission among dogs. Despite regular *parasite prevention* and cleaning measures in these shelters, previously infected dogs may retain *Ehrlichia*, underscoring the challenge of eradicating the infection entirely.

The data analysis results indicate that the dog’s gender does not influence *E. canis* infection, mirroring findings from previous studies. For example, a retrospective study involving 100 dogs with canine monocytic ehrlichiosis in Israel demonstrated no gender predisposition for monocytic ehrlichiosis occurrence in dogs [27]. However, a canine ehrlichiosis serological test carried out in a rural area of Brazil revealed a higher positivity rate among male dogs, conflicting with our research outcomes [25]. This discrepancy in results may be attributed to the behavioral traits of male dogs, known for being excitable, active, and engaging in broader activities during the estrous period, thereby increasing their exposure to ticks compared to female dogs.

Our research findings indicate that dogs in Hainan province, particularly rural and stray dogs, are vulnerable to *Ehrlichia* infection. While *E. canis* primarily infects dogs and there are no documented cases of human *Ehrlichia* infection in China, human infections with *E. canis* have been reported in Venezuela, Panama, and Northern Mexico [28–30]. This suggests that tick vectors indeed have the potential to transmit *Ehrlichia* to humans and are pathogenic to them, posing a public health threat to both dogs and humans in Hainan province. These findings contribute new insights into China’s epidemiological database of canine vector-borne diseases. They will aid in developing effective measures to safeguard the health of companion animals and their owners. The perpetual warm and humid tropical climate conditions in Hainan province create an ideal environment for tick survival and reproduction. Additionally, as a tourist island and free trade zone in China, Hainan province experiences significant annual movement of people and pets. These factors elevate the risk of rapid transmission of zoonotic tick-borne diseases on Hainan Island, constituting a substantial factor for One Health. Consequently, these factors should be prioritized when formulating tick-borne disease prevention and control measures for Hainan Island.

The concept of One Health has garnered support from numerous medical and veterinary associations, with a critical focus on curbing the spread of infectious diseases. Within the realm of One Health, companion animals hold significant potential. The bond between humans and their companion animals is deepening, and healthy animals contribute to human well-being by mitigating the transmission of zoonotic diseases to some extent. The significance of zoonotic parasitic diseases in One Health is increasingly recognized, particularly the emphasis on tick-borne diseases. Presently, the geographical range of ticks is expanding due to climate variations, economic globalization, and other bio-geographical factors, amplifying the threat posed by tick-borne illnesses. As economies evolve and pet ownership rises globally, companion animals assume a crucial role in family dynamics. While many companion animals receive high-quality medical care, they can still serve as hosts for various tick-borne diseases. Furthermore, in economically disadvantaged regions or nations, there exists a substantial population of stray dogs and cats that interact closely with urban environments. Despite limited human interaction and minimal veterinary care, these stray animals remain significant reservoirs for tick-borne diseases.

## Conclusion

In the present study, we investigated the epidemiology of *Ehrlichia* spp. in dogs and ticks from Hainan, China, utilizing PCR. A total of 631 tick samples were collected from dogs in Hainan province, all of which were identified as *R. linnaei*. Among them, 63 tick samples (9.98%) tested positive for *Ehrlichia* spp., all of which were *E. canis*. Furthermore, we collected 1,264 canine blood samples from Hainan Island and detected 140 cases (11.08%) of *Ehrlichia* infection, all identified as *E. canis* infection only. Notably, the sequences of *E. canis* from both dogs and ticks displayed high homology. The *E. canis* sequences from Hainan province formed a cohesive cluster closely associated with *E. canis* sequences identified in Mexico (OP268413.1) and Brazil (KF972450.1). Our investigation revealed that *E. canis* infection in dogs is related to various factors, including age, breed, anthelmintic usage, tick infestation, and living environment.

## Data Availability

All data generated or analysed during this study are included in this published article.

## Abbreviations

E.canis: Ehrlichia.canis
E.ewingii: Ehrlichia. ewingii
E.chaffeensis: Ehrlichia. chaffeensis
R.linnaei:Rhipicephalus: Linnaei R.sanguineus
Rhipicephalus;A.americanum: Amblyomma.americanum
HME: Human Monocytic Ehrlichiosis
PBNS: Peripheral Blood Neutrophils
CGE: Canine Granulocytic Ehrlichiosis
